# Social Media and Health Care, Part I: Literature Review of Social Media Use by Health Care Providers

**DOI:** 10.2196/23205

**Published:** 2021-04-05

**Authors:** Deema Farsi

**Affiliations:** 1 Department of Pediatric Dentistry Faculty of Dentistry King Abdulaziz University Jeddah Saudi Arabia

**Keywords:** social media, social networking, internet, health care, COVID-19, research activity, medical education, telemedicine, mobile phone

## Abstract

**Background:**

As the world continues to advance technologically, social media (SM) is becoming an essential part of billions of people’s lives worldwide and is affecting almost every industry imaginable. As the world is becoming more digitally oriented, the health care industry is increasingly visualizing SM as an important channel for health care promotion, employment, recruiting new patients, marketing for health care providers (HCPs), building a better brand name, etc. HCPs are bound to ethical principles toward their colleagues, patients, and the public in the digital world as much as in the real world.

**Objective:**

This review aims to shed light on SM use worldwide and to discuss how it has been used as an essential tool in the health care industry from the perspective of HCPs.

**Methods:**

A literature review was conducted between March and April 2020 using MEDLINE, PubMed, Google Scholar, and Web of Science for all English-language medical studies that were published since 2007 and discussed SM use in any form for health care. Studies that were not in English, whose full text was not accessible, or that investigated patients’ perspectives were excluded from this part, as were reviews pertaining to ethical and legal considerations in SM use.

**Results:**

The initial search yielded 83 studies. More studies were included from article references, and a total of 158 studies were reviewed. SM uses were best categorized as health promotion, career development or practice promotion, recruitment, professional networking or destressing, medical education, telemedicine, scientific research, influencing health behavior, and public health care issues.

**Conclusions:**

Multidimensional health care, including the pairing of health care with SM and other forms of communication, has been shown to be very successful. Striking the right balance between digital and traditional health care is important.

## Introduction

### Background

A key characteristic of being human is the ability and desire for social networking. Over the ages, humanity has thrived in social communities in which members shared knowledge, opinions, and experiences, empowered by a sense of belonging. As the world continues to advance in terms of technology, social media (SM)—defined as “a group of Internet-based applications (apps) that allow the creation and exchange of user-generated content”—is becoming an essential part of billions of people’s lives worldwide and is affecting almost every industry imaginable [1]. The definition of SM (the “read, write web,” “Web 2.0,” or “social networking”) is constantly evolving [2]. The Merriam-Webster Dictionary defines it as “any form of electronic communication through which users create web-based communities to share information, personal messages, ideas, and other content such as photos and videos” [3]. SM is considered one of the most powerful communication tools of the 21st century. There has been a proliferation of SM tools in recent years, creating new opportunities to communicate, connect, create, and share information, without requiring exceptional coding skills to create or retrieve content [4].

Specifically, SM is increasingly becoming an augmenting tool in health care by enabling its users to acquire and share information; connect with others in the field; and communicate with colleagues, patients, or the public regarding health topics. Furthermore, SM supports patient empowerment by expanding the knowledge of the patients and placing them in a position where they can take control of their own health care needs [5]. This review is based on numerous studies and reviews that have investigated the different uses of SM in health care and its limitations and shortcomings. Consequently, this narrative is comprehensive and up to date, including the recent use of SM during the COVID-19 pandemic. The topic is relevant in today’s scenario because the use of SM and social networking sites (SNSs) is increasing worldwide, especially in the health care industry. The findings presented in this review have strong implications for health care professionals, educators, and researchers.

### Objectives

This review aims to shed light on SM use worldwide and discuss how SM has been an essential tool in the health care industry from the perspective of health care providers (HCPs). The review will be continued in Part II, where the use of SM from the perspective of patients will be discussed.

## Methods

### Search Strategy and Information Sources

Between March and April 2020, a comprehensive search on 4 databases (MEDLINE, PubMed, Google Scholar, and Web of Science) was conducted for all English-language medical studies that were published since 2007 and discussed SM use in any form for health care. A combination of the following keywords was used to search for titles and abstracts: “social media” (MeSH term) OR “social networking” OR “internet” (MeSH term) OR “WhatsApp” OR “Instagram” OR “Facebook” OR “YouTube” OR “Twitter” OR “LinkedIn” AND “healthcare” OR “health” (MeSH term) OR “medicine” (MeSH term) OR “physician” (MeSH term) OR “nursing” (subheading) OR “dentistry” (MeSH term) OR “telemedicine” (MeSH term), “recruitment” OR “education” (subheading) OR “career” OR “behavior” (MeSH term) OR “research” (MeSH term). Each of the 9 words in the first set was separately searched with each of the 12 words in the second set using “AND.”

### Screening Process

The articles were entered into an EndNote library, and duplicate publications were removed. Articles published before 2007 were excluded, as the words *social* and *media* at that time did not represent the current definition of SM. Titles and abstracts were assessed for eligibility. Studies that were not in English were excluded, along with those with inaccessible full text after unsuccessful attempts to access them. Irrelevant studies, such as studies that were not related to health care, studies whose primary outcome was not the use of SM in health care, or studies that discussed the negative impact of SM on health, were also excluded. Dissertations were also excluded from the study. The full texts of the studies were then appraised. Several relevant studies investigating SM use from patients’ perspectives were found. Reviews on legal and ethical issues pertaining to the use of SM in health care were also obtained, following which, the publications were divided into 4 groups: *HCP*, *patient* or *the public*, *ethics* and *legal considerations*, and *shortcomings*. A decision was made to defer reviewing the last 3 groups and focus on this review on SM use by HCPs.

### Categorization

After accessing the complete texts of the articles of interest, their reference lists were searched for additional studies, and the cited studies were also located. Thereafter, the articles were comprehensively reviewed. On the basis of the key findings, articles were initially grouped as follows: *sharing information*, *recruitment*, *education*, and *marketing*. As the review proceeded and more information was obtained, the groups were modified. *Sharing information* was divided into 2 groups: *health promotion*, focusing on HCPs sharing scientific information with the public, and *critical public health care issues*, which focuses on health announcements in crisis, especially COVID-19–related publications that warrant special attention. *Recruitment* was also divided into 2 groups: *recruitment*, which included job employment and residency program enrollment, and *scientific research*, in which studies discussed recruiting research participants and analyzing SM data. *Education* was renamed *professional medical education*, as this name specifies medical education. Studies related to continuous education were added to *marketing*, and the group was renamed *career development* and *practice promotion*. Another group was created—*professional networking* and *destressing*—which included findings from *sharing information* that discussed peer-to-peer communication and those from *education* that did not reflect professional education or career development. Finally, an additional group was created, *telemedicine*, as studies on this subject were abundant.

## Results

### Summary and Characteristics of Included Studies

**Figure 1:**
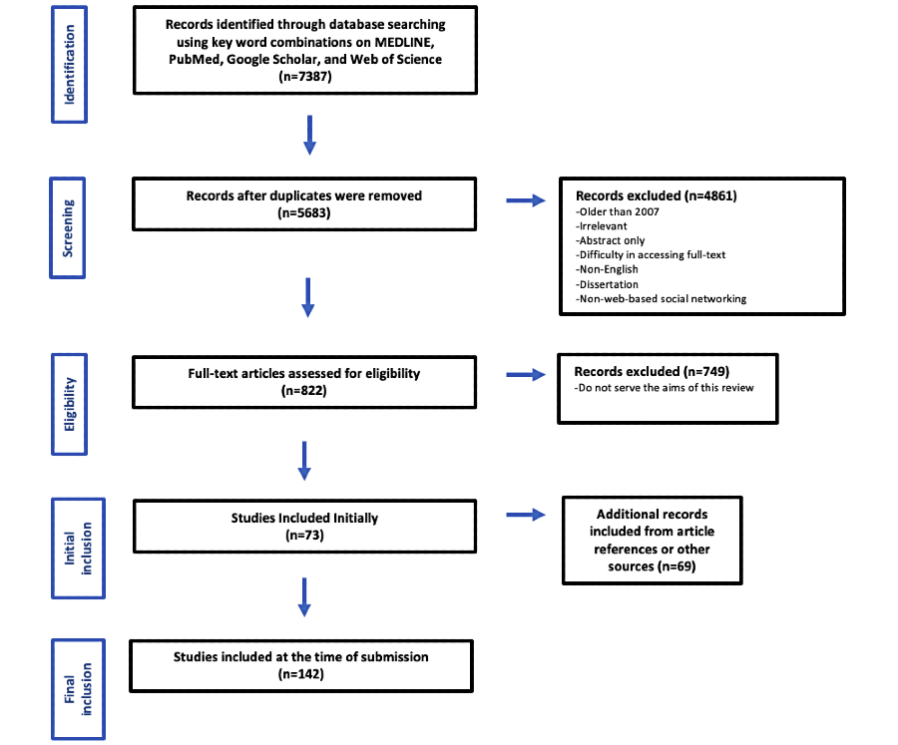
Flowchart of the selection procedure.

The search yielded 5683 titles that were scanned with their abstracts. After exclusion of duplicates and noneligible studies, the initial sample comprised 73 publications. The full-text papers were retrieved. Additional studies from the article references or those emerged from the review but were not identified earlier were also added. This was because of variation in the keywords with respect to spellings (eg, behavior and behaviour), terminology (social networking and social network), and synonyms (eg, recruitment and employment) that were not accounted for in the initial search. A total of 142 articles (63 original studies) and 3 textbook chapters were reviewed (Figure 1).

**Figure 2:**
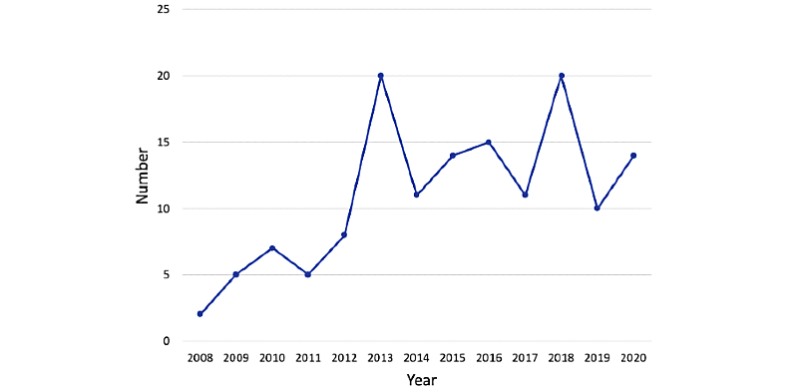
Number of included publications per year.

The studies were conducted in the United States (61), Canada (12), Brazil (2), the United Kingdom (12), Europe (22), the Middle East (9), India (9), Asia (8), and Australia (7). The earliest study was published in 2008, and the latest studies were published in 2020, with most of them being published after 2014 (Figure 2).

### SM Platforms

Most reviews discussed SM in general and did not specify a particular platform; however, some original studies investigated specific platforms. The platforms investigated were WhatsApp/WeChat (15), Facebook (8), Twitter/Weibo (9), YouTube (4), Instagram (1), LinkedIn (1), Podcast (1), and Wikipedia (1).

### Medical Specialties

Some reviews discussed SM use in a particular medical specialty, whereas others reviewed studies from diverse or unspecified specialties. Researchers from a variety of medical specialties investigated SM use in their original studies. These specialties were surgery (1), plastic surgery (4), neurosurgery (1), maxillofacial surgery (2), medicine (2), emergency medicine (2), psychiatry (3), orthopedics (3), otolaryngology (1), immunology (2), dermatology (1), radiology (1), urology (2), anesthesia (1), cardiology (1), pediatrics (1), oncology (3), nursing (5), dentistry (11), and pharmacy (1).

## Discussion

### Principal Findings

This literature review aimed to examine SM use in the modern world and how it has been recently incorporated into health care. Most of the reviewed articles were published in the past decade, suggesting that this review is both relevant and contemporary. It is evident from the published studies that SM has broad applications in modern health care. As discussed in the subsequent sections, HCPs (the term is used in this review as including physicians, dentists, nurses, medical and dental allied personnel, and health care organizations) not only use SM to provide care to their patients but also for personal development and destressing.

### SM Use

SM use is one of the most common web-based activities, with an estimated 2.9 billion users worldwide as of 2019, a number that is projected to increase to 3.4 billion by 2023 [6]. With this, digital networking has witnessed a massive growth, and social communities have become boundless. Facebook, Twitter, Instagram, WhatsApp, and Google are relatively new platforms, but they are being used every day by millions of people worldwide. SM platforms are among the most commonly used sources for acquiring and disseminating information [7,8]. They are not only used for socialization, knowledge acquisition, and entertainment, but they have also been linked to significant political events led by young users [2].

Many SM tools have been introduced, and they continue to evolve. They may be categorized as tools for social networking (eg, Facebook and Instagram), professional networking (eg, Doximity and Sermo), media sharing (eg, YouTube and WhatsApp), content production (eg, Twitter), and blogs [9].

In terms of technological knowledge, SM users belong to 1 of the following 2 groups, as classified by Prensky [10]: digital natives and digital immigrants. Digital natives are those born after 1980, who are skilled in using technology, and who rely heavily on technology and social networking. Most digital natives were introduced to technology at an early age. Conversely, digital immigrants are those who acquired technological skills and adopted technology later in their careers [9]. SM use is generally high among digital natives, who explicitly prefer it over traditional media [11]. Some researchers believe that there is no dichotomous divide between internet users and nonusers. Although the terms are commonly used, Prensky’s model and its usefulness have been challenged [12]. First, basic digital skills are not difficult to acquire, especially with repetitive use. With practice, a person born in the 1960s can become as digitally fluent as a millennial. Second, the distinction between both generations implies that digital immigrants can never completely acquire digital abilities and that digital natives are automatically technologically skilled. This approach is neither scientific nor based on any empirical evidence. Third, the model overlooks the fact that age is not the only factor in determining digital skills. Socioeconomic and cultural factors of digital capability must not be ignored. For instance, a millennial who lacks access to technology is not a digital native.

Regarding SM demographics, its use is prevalent across all ages and professions [13]. However, different SM platforms differ in their demographics. The demographics of some of the most commonly used SM platforms worldwide can be further explored. Facebook has 2.7 billion monthly active users. According to a 2020 report, the highest number of Facebook users were aged between 18 to 29 years and 30 to 49 years, with more female than male users and more urban than suburban or rural users [13]. India had the largest number of users, followed by the United States, Indonesia, and Brazil. Regarding Instagram, there are 1 billion users globally. As of August 2020, there were more female than male users, and the United States had the highest number of users [13]. Users aged between 25 and 34 years represented the largest group of users [14]. Twitter had over 330 million users, who were predominantly male [13]. The top 3 countries for Twitter use were the United States, Japan, and India [13]. Approximately 30% of all users were aged between 25 and 34 years [15]. The Chinese Twitter-like SM platform is Weibo, and it had an estimated 480 million users [16]. WhatsApp is a mobile messaging app that is used by 2 billion users in 180 countries and in 60 different languages [17]. WhatsApp is more commonly used by younger people [17]. WhatsApp’s direct Chinese competitor is WeChat, and it has about 1.17 billion users [18]. YouTube is commonly used worldwide, particularly in the United States. It is estimated that it has approximately 2 billion users. The users are more commonly male than female, and its use is prevalent in urban, suburban, and rural locations [13]. Finally, there were about 46 million students and recent college graduates on LinkedIn out of 675 million users [19]. Male users constituted 67% of the total users, and the United States had the highest number of users, followed by India, China, and Brazil.

Although most SM platforms share common features such as free registration, public and private communication, and fast content upload and retrieval, each platform is unique and has distinctive uses. It is common for users to have different accounts across multiple platforms, using each platform for different purposes. Facebook is an SNS that can be accessed from any internet-enabled device, such as personal computers and smartphones. Registration on Facebook is free, and users can create a profile that reveals selective information about themselves [20]. Users can post text, photos, and multimedia that become available to any user in their *friend list*. Users generally begin by adding family members and friends to their friend list, which can be expanded to include colleagues, acquaintances, and strangers with or without common interests. Apart from being able to share public comments and *likes*, a few years after Facebook was founded, a messaging feature was added that allows users to send private messages to individuals and groups. Users can use a variety of embedded apps; join and create *groups* and *pages*; play games; and receive updates regarding the activities of their friends, pages, and groups. Although the platform was initially limited to students in certain American universities, Facebook now has users worldwide. Instagram is a newer SM platform owned by Facebook and is designed primarily for free photo and video sharing [21]. By modifying their privacy preferences, Instagram users can opt to have either public accounts or limit their content to users that they accepted as *followers*. The platform also allows viewing, commenting on, and *liking* posts shared by users that they follow as well as private messaging between users. WhatsApp, which was acquired by Facebook in 2014, is a text and voice messaging app that has become incredibly popular owing to its features, flexibility, and compatibility with various phone and computer operating systems [22]. Although a free service, WhatsApp allows exchange of messages and calls on both desktop and mobile devices, in addition to media sharing and group features. WhatsApp’s objective was to provide an alternative to SMS. Using WhatsApp, billions of users across the globe can simultaneously and instantly connect with others.

“Twitter is what’s happening in the world and what people are talking about right now”—this is how Twitter describes itself [23]. It is a microblogging platform that allows users to post and access short text, image, or video posts called tweets. Although tweets were originally limited to 140 characters, the limit was increased to 280 characters in 2018, along with permitting the sharing of website links and multimedia. Twitter’s mission is to provide users with the ability to create and share ideas and information instantly and without barriers. Users follow other personal, official, or organizational accounts. They can either create their own tweets or *retweet* those by others to their followers. YouTube is a web-based video-sharing platform that allows users to upload, view, share, rate, report, comment on videos, and subscribe to other users [24]. Its mission is to provide users with a voice through video sharing, stemming from the belief that the world would be a better place when people listen, share, and build a community through their stories. The name of this platform is straightforward: *You* represents content that is user-generated and not created by the site itself, and *Tube* is an older term for television. Most YouTube content is uploaded by individuals, but some media corporations have established partnerships with YouTube to offer some of their materials on this platform. LinkedIn, acquired by Microsoft in 2016, is a business and employment-oriented SM service that operates as both a website and mobile app. LinkedIn is mainly used for professional networking, allowing employers to post about job openings and seekers to share their curricula vitae [25]. Using the platform, users can build strategic professional relationships rather than expand their friend circle. LinkedIn’s vision is to provide professional opportunities to its users, and its mission is to connect professionals worldwide. It originated in the living room of one of its cofounders a year before its launch in 2003. LinkedIn today has a diversified business model that has generated successful recruitments.

SM users have claimed that they have more digital friends and connections than real-world ones, which highlights the transformation of the ways in which people connect with each other and the importance of web-based relationships in today’s world [26]. Over time, social networking platforms have targeted different age cohorts, making SM use widespread among the general population. For example, as of April 2020, men aged between 25 and 34 years constituted the largest demographic group of Facebook users, and those aged 65 years and older were the fastest-growing group [27,28]. Similarly, the largest group of Twitter users were people aged 25 to 34 years, whereas 15% of users were older than 50 years [15].

The public attitude toward SM use has drastically changed over the years as it became more accessible and diverse in its offerings. Consequently, SM has become a universal communication channel, and responses in reality and on the web have increasingly become intertwined and concurrent [29,30]. Furthermore, SM offers lucrative opportunities to disseminate information and thoughts directly to the public, share experiences, build communities, and connect people with common interests, something unthinkable 20 years ago [31].

### SM Use in Health Care

The more digitally oriented the world becomes, the more the health care industry visualizes SM as an important channel for health care promotion, employment, recruitment of new clients or patients, marketing for HCPs, and building a captivating brand name. HCPs have realized that SM is not just a platform to post vacation photos and interact with followers. Perhaps the 4 most common areas where SM plays a major role in the health care industry are health promotion, research, marketing and branding for individuals and practices, and recruitment. It has been evident that web-based content can be spread to offline environments, such as classrooms and meeting rooms. Furthermore, SM has undeniably changed patient-practitioner relationships because of patients’ better understanding of health information and their more active role in health maintenance [32].

The effect of behavioral and social factors on health outcomes has evolved significantly in recent decades [33]. HCPs continually search for new and more efficient methods to reach larger populations, especially those who were inaccessible via traditional methods. It is incumbent upon them to use every available tool to reach their intended audience. Thus, HCPs and health organizations should capitalize on the opportunities provided by SM and update strategies to reach communities and age cohorts at a relatively low cost [7,34]. In other words, SM brings a new dimension to health care and is changing the nature and speed of health-related interactions between individuals and health organizations. For example, communicating through photos and videos along with text is part of the mobile revolution, and messaging apps are now regarded as a viable medium for sharing knowledge and discussing clinical cases [35]. In summary, using SM could be a key strategy in addressing some of the challenges and limitations often faced by HCPs in traditional health communication through faster and cheaper dissemination, more accessibility, better interaction, and increased patient empowerment [7]. Moreover, information can now be easily brought to audiences with special needs or low literacy [36].

In the fast-paced modern world, time constraints are common in medical practice, and when combined with the demands of individuals with chronic conditions or unplanned emergency situations, it is challenging for HCPs to dedicate extra time to patients. SM can provide efficient and easy-to-use platforms that encourage patient-practitioner interaction and facilitate necessary actions from both ends [37]. In fact, as of April 2018, there were more than 200,000 health apps, including social networks for people living with a specific medical condition; approximately 19% of smartphone users have at least one health app on their phones [38]. Furthermore, in a 2013 survey of more than 4000 physicians, 65% stated that they used SM for professional reasons [39]. SM use has not been limited to HCPs. Patients have also incorporated SM in their daily lives, which encourages HCPs to explore different ways of making their mark in this growing market [31].

Technology is evolving very rapidly [40]. Competition produces better services, and the diversity of options enables users to choose a tool that best matches their individual needs. Although different platforms often have different target demographics, audience overlapping may occur and should be considered by health organizations when devising their SM health promotion strategies. In health care, SM tools can be used for different purposes (health promotion; dissemination of health information; education; professional development; recruitment; communication with the public, colleagues, and patients; and research) and in diverse medical specialties (cardiology, nursing, radiology, dentistry, surgery, pathology, pediatrics, pharmacy, emergency, and critical and palliative care) [41]. As of August 4, 2020, 27,546 results appeared when searching for *social media* on PubMed, demonstrating the growing interest in SM within the health care industry.

Health care systems, especially in times of crisis and outbreaks, require the dissemination of information to practitioners, patients, and the general public rapidly and effectively [34]. Health organizations and officials, by taking upon a more active SM presence, gain access to vast global networks capable of quickly spreading information and promptly mobilizing large numbers of people toward public health goals [2,42]. Never before has the entire world united as it has in recent months in its fight against the disease caused by SARS-CoV-2, more commonly known as COVID-19. Searching *COVID-19* on PubMed on August 4, 2020, yielded 37,576 results, which exposes the abundance of information and data that has become available in 7 months since the beginning on the pandemic [8]. However, the World Health Organization (WHO) has expressed concerns about fighting 2 battles at once: the pandemic and the *infodemic*—the latter refers to a rapid and far-reaching spread of both accurate and inaccurate information about the disease [43].

It is important to mention that the popularity of SM is directly linked to its many advantages. Advantages of using SM in health care include its expressive nature, accessibility from a smartphone, prompt content sharing and response generation, improved and two-sided communication, reduction of consultation time, smoothing of hierarchy, more efficient teamwork, ability to forge connections between people, and ability to reach large masses [44,45]. Furthermore, SM facilitates the access to health information for extended population groups, regardless of geographic location, age, or education, compared with traditional communication methods [41]. However, the most influential advantage of SM remains its cost-benefit feature: it can reach an increasing number of people without the high cost of traditional means and the information remains available 24 hours a day, 7 days a week.

Similar to most technologies, SM has its disadvantages. In the health care industry, these include increased workload, risk of unprofessional behavior, disparity in the sense of urgency, a demanding sense of needing to stay connected 24 hours a day, difficulty in obtaining discussion records, worries about leading to or identifying patients, privacy breech, change of patient-practitioner relationship from a professional to a personal one, and the risk of reducing the autonomy of junior doctors [45]. Those who choose to use SM should be aware of the potential risks and problems that they could encounter but should not shy away from using SM because it can greatly increase the reach and impact of HCPs’ work and improve patients’ health [34]. In the following section, the specific uses of SM in health care are discussed in more depth.

### SM Use From the Perspective of HCPs

The literature review yielded an abundance of information. The studies were categorized as follows to best present the findings: health promotion, career development or practice promotion, recruitment, professional networking and destressing, professional medical education, telemedicine, scientific research, and critical public health care issues.

#### Health Promotion

Producing and disseminating information has played a pivotal role in the history of humanity. Over the years, an increasing number of public health organizations, medical institutes, and HCPs are using SM tools to disseminate visually rich public health messages to the general public. The primary goal is to share solid, evidence-based, and up-to-date health information that educates and affects millions of SM users and to dispel common misconceptions and counterbalance inaccurate material rapidly spreading through SM [2,32]. Examples of how SM can increase the accessibility of a massive number of recipients to health care information around the world include concise educational tweets on Twitter, a pediatric dentistry group on Facebook where fellow specialists discuss anonymized cases and share ideas, photos of a salvation mission to an underprivileged community on Instagram, and the results of a clinical study broadcasted via WhatsApp. These are all examples of how SM can not only increase accessibility, but it can do so at a faster rate than any other means, and perhaps in the cheapest way possible.

Access to oral health care services is limited by a lack of universal coverage. SM, which is a method of mass communication, offers an alternative to traditional communication, which extends to reach underprivileged and underserved communities. The WHO and the United States Centers for Disease Control and Prevention (CDC) are among many other public health institutions that use SM to communicate with the public during public health crises and natural disasters [8,29,35]. Physicians also use SM to promote patient health care education on a smaller scale within their networks. Research has shed light on the many tools that have been used for this purpose. For example, HCPs can tweet, record videos, and participate in health-related discussion forums, which provides an opportunity for physicians to share scientific information and broaden their knowledge [46,47]. Furthermore, information from international conferences and findings from the latest research and clinical trials can be presented in mainstream media to be shared with millions of people [48].

Sharing such information not only helps improve knowledge but can also improve attitudes and practices related to health. For example, in dentistry, SM has played a role in helping patients cope with challenges such as dental anxiety and in presenting dental management options in a convenient and nonthreatening manner [42,49]. Evidence now shows that SM-based interventions are linked to healthy practices such as tobacco cessation, increased physical activity, and diversion from risky sexual behaviors [39,50].

In conclusion, there is evidence that SM helps to improve access to health information. When designing SM campaigns and interventions to disseminate health information, it is important to develop messages that may be more likely to resonate with and elicit reactions from individuals [2]. Messages tailored to certain population segments are more effective than generic messages, as tailored messages address the specific needs of their recipients [51]. Furthermore, interactive (two-way) communication is more effective than linear (one way) communication [7]. Importantly, SM must complement rather than replace traditional health promotion. More research is needed to investigate strategies that can increase access to health information for minorities and marginalized communities and for populations deprived of internet access.

#### Career Development or Practice Promotion

One of the measures of the success of HCPs is their ability to attract and retain patients. This will not only maximize income but will also boost reputation. SM has played an important role in enhancing practice or practitioner ranking on search engines, even more than academic pedigree and experience [52]. As search engines generally direct patients’ traffic, a strong presence on the web can be crucial to attract patients to a practice.

HCPs at all stages of their careers can use SM to brand their name. SM aids in developing their name, expanding their network, and learning about career-enhancing opportunities [53]. It can also be used as a marketing strategy to attract patients of various demographics and has been proven to be effective in engaging and obtaining new patients [54]. In a survey conducted in 2013, 12.5% of health care organizations reported attracting new patients through SM [39]. Moreover, a 2012 study of dental practices in the United States revealed that 51% of the practices used SM, of which 91% used it for marketing purposes and 73% used it to increase their presence on the web [55].

With the extended use of SM among patients and HCPs, practitioners must now compete for patients’ attention and need to be strategic regarding the content they share and platforms they use [7]. HCPs should advertise their professional trajectories, areas of experience, and treatment outcomes by focusing on information tailored to the target audience in an educational manner that does not typify commodification or unfair competition. The eagerness to achieve popularity and to attract new customers or compete with colleagues results in some HCPs thinking only with a short-term approach and prioritizing greater financial gains. For example, some orthodontists and plastic surgeons post before-and-after photographs with drastic improvements without explaining that biological variations among patients, differences in response to treatment, and other external factors may affect the course and outcome of the intervention. Without such information, patients tend to have unrealistic expectations and end up being disappointed [56]. Unfortunately, some practitioners tend to digitally modify images to accentuate treatment-led improvements. This misuse of technology could lead to serious reputational damage for the practitioner and the profession in general in addition to unfavorable court decisions [56].

HCPs build their status using SM in diverse ways. They begin by creating a profile page on one or multiple platforms, which allows them to create and upload content. By connecting with colleagues, they can begin to establish a digital social network. Moreover, groups based on common interests further expand their social network and raise the practitioner’s name in bigger circles. These processes can create a haven for viral marketing, which can be leveraged to create a name, develop a digital voice, and disseminate health information in a timely and cost-efficient way [7,30,42]. Moreover, for newly qualified practitioners, contributing to discussions on forums and virtual meetings raises their profile among more experienced practitioners who may be geographically distant. This can leverage word-of-mouth referrals and attract fellow researchers to collaborate.

In terms of cost-effectiveness, a 2017 study on the SM return on investment (ROI) showed an upward trend that represented stable growth for Facebook, whereas Instagram demonstrated substantial ROI. It was concluded that SM resources were superior to standard internet-based resources. When all SM platforms were combined into one graph, there was a consistent finding of growth associated with all SM sources over time [57]. As the following quote summarizes, “physicians have to realize that our patients are doing it (SM), so this is where we need to be” [58].

In summary, there is no one-size-fits-all SM platform, and there is no single way to share content that is superior to all others. It is essential for an HCP to emphasize the importance of their specialty; present the strengths in their particular practice; understand the features and user demographics for SM platforms; and, most importantly, know their target audience. For example, a plastic surgeon or orthodontist may find it beneficial to share pre- and posttreatment or procedure photos of anonymized patients, for which Instagram may be the ideal platform. A practice that is community friendly may invest in a Facebook page to keep the audience updated on offers and services. A family medicine office may share announcements regarding the arrival of a flu-vaccine and post photos of staff members vaccinating themselves to motivate people. Twitter may be useful for posting specialty-related educational messages or sharing information on health-related matters to make HCPs more visible. A pediatric dentist may use YouTube to share videos of tricks used in the office to make the experience less threatening for children. More training courses and talks on how to leverage SM to establish a presence and build a name may be beneficial for HCPs who are not SM savvy.

#### Recruitment

SM is making great strides not only in the modern world of technology but also in the workplace: it is transforming the way people find and engage in work. It seems that the conventional channels for recruiting employees are not as effective as they once were. Instead, we are shifting toward SM not only as a platform for social interaction, photograph exhibition, and creative expression but also as a space for far-reaching, low-cost job searches. Regarding employment, the interest in SM is bidirectional. On the one hand, employers are often keen to know more about a candidate applying for a position than what is stated in their resumé. On the other hand, employees, especially millennials, will first want to know more about the dynamics of a firm and the personalities of their future boss and coworkers before they commit to the job. Recruitment in the medical field requires more than an application. In this section, the recruitment of HCPs for employment or students for residency programs is discussed.

Human capital is of major importance to any organization because humans produce income and are a source of competitive advantage [59]. Recruitment of qualified employees who are fit for the job is not a simple, one-way decision as it used to be. Performing due diligence in hiring a new employee is more essential than ever and is a multidimensional process, including at least one interview, drug screenings, and background checks. More recently, employers have turned to nontraditional methods and to SM to further analyze potential candidates [60]. SM prescreening may have the potential to offer information about the applicant above and beyond what is stated in the resumé and can be assessed in a more traditional screening [61]. For job seekers, because of the large number of SM users and the relatively low cost of setting up, SM platforms are ideal for finding employment. Furthermore, many organizations are now investing in SM to display their employer brand and, in return, attract qualified applicants [59,62]. Organizations aiming to attract applicants, especially in fields where competition and demand are high, such as in nursing, must make every effort to promote a unique brand image and attract potential candidates [59,63].

There is evidence that recruiters can accurately determine productivity-related traits solely on the basis of personal information about a candidate available on SNSs [64]. Baert [65] found that personal photographs have become more effective as objects of communication than of memory. This research described interesting theoretical mechanisms that underlie better labor market outcomes for more attractive people. For example, it proposed that self-confidence from good looks could drive productivity, leading to emotional stability, and, consequently, labor market success [66]. The study also found a higher impact of face pictures seen on Facebook’s profile photo compared with those attached to a resumé [65].

From a job seeker’s point of view, SM makes it possible to apply to hundreds of jobs, even globally, at once. Through SM, job applicants can increase their presence on the web to grab the attention of employers [60]. In the health care industry, it is advisable for job seekers to be active in various medical societies to expand their connections and to make a positive impression on future employers. Similar to the real world, it is advisable for applicants to attend virtual conferences and discussion boards and to introduce themselves to others at every reasonable opportunity [67]. It is important to note that employers do not use only professional platforms such as LinkedIn for hiring. In fact, they check many SM platforms when screening for prospective employees [60]. It is not a bad idea that employers and applicants conduct periodic searches for their own names to ensure that their SM persona projects a professional image [2].

Several studies have investigated the effects of SM on recruitment in health care. It was found that a hospital’s profile on SM can shape employer brand perceptions and attract nurses. In addition, nurses who visited the hospital’s Facebook page were more attracted to work there [59]. In another study, over 92% of employers stated that they were planning to use SM for recruiting [68]. Moreover, a study conducted in 2012 found that the recruitment and screening costs were reduced by 50% by using SM and that 65% of employers were evaluating the integrity and character of potential employees based on their SM profiles [60]. As shown in a review by Davison et al [61], a study found that 20% of the organizations surveyed were planning to use SM for applicant screening. Furthermore, LinkedIn was the most commonly used SM platform for screening applicants, whereas the use of Twitter for screening purposes increased from 11% to 31% between 2011 and 2013 [61]. Interestingly, a study found that there were 38% more job interview invitations to candidates with the most beneficial Facebook pictures [65]. In addition, a Microsoft survey revealed that 79% of employers searched for web-based information regarding prospective employees, but only 7% of the candidates were aware of this possibility [69,70].

Regarding residency programs, SM is a mechanism to accentuate the programs’ visibility on the web and to screen residency applicants [71]. It is important to note that it is not only credentials and high scores that secure a spot in a specialty program; personality traits and characters are becoming more significant than ever. Program directors (PDs) now want to know applicants on a personal level. As much of this information would be illegal to obtain in a traditional interview, they may search for it on SM [72]. Admission officers and PDs are now capitalizing on the abundance of information and the popularity of SM [72]. They may encounter content that seems unprofessional or exposes negative character traits that are useful in making decisions about applicants. Many residency programs now search Facebook and other personal SM platforms to screen applicants [73]. Even residents are now using SM platforms to obtain information on possible postgraduate opportunities [71].

There is an abundance of research on the use of SM for applicant selection in residency programs. In one study, 17% of PDs screened applicants on SM, 33% gave lower rankings to applicants based on SM findings, and 69% stated that they will continue to use SM for applicant screening [74]. In another survey, most school children who were interested in studying medicine felt that behaviors on SM should be considered for admission to medical schools [75]. Furthermore, a study conducted in 2016 found that 18% of PDs visited the SM profiles of residency applicants, 10% gave a lower rank or completely disqualified an applicant because of negative web-based behavior, and 10% took formal disciplinary action against a resident because of negative web-based behavior, with Facebook being the platform used by most PDs [76,77]. Another survey found that 97% and 90% of PDs agreed that candidates should be held accountable for illegal acts and unprofessional behavior on the web, respectively, whereas 89% of them agreed that information voluntarily published on the web is fair to use in judging character and professionalism. Furthermore, 82.4% of PDs indicated that they would favor the candidate with a sterile Facebook profile if they were choosing between 2 mock candidates [78]. Moreover, student pharmacists demonstrated a general attitude that web-based personas on SM should not be used to judge professional attitudes and abilities [79]. Although most medical school PDs believed that screening applicants on SM does not constitute a violation of the applicants’ privacy, the topic remains controversial and views regarding the appropriateness of using SM profiles to judge character and professionalism vary [78,80]. There is general agreement that SM information is open for judgment by others, especially among older PDs [78].

Professionalism is advocated by the American College of Surgeons as a quality that extends beyond the clinic, operating room, and hospital and into the community in the real world and on the web [81]. There are some issues associated with using SM to judge a possible employee’s or resident’s professionalism and character. First, screening is usually done by a single person without a standardized scoring rubric. Second, content is unstandardized among the different SM platforms, and the information displayed differs across platforms; for instance, it would not be fair to compare someone’s Facebook photo album of a Spring Break trip with someone’s contribution to a medical discussion on Twitter. Moreover, screening SM content showed poor test-retest reliability, especially as the content could change rapidly. Interrater reliability is potentially affected by the content being rated and the characteristics of the rater. Construct validity also seems to be weak as no specific construct is usually in mind; instead, a rater casually scans profiles to make a judgment on an applicant or screen potential new hires. Finally, there is a problem with generalizability across platforms. It is suggested that personality traits should be judged from platforms with flexible formats (eg, Facebook), whereas professional traits and experiences should be judged from more structured platforms (eg, LinkedIn) [61].

In summary, although e-professionalism is a new topic, it is receiving considerable attention from recruiters and is being taught as a part of medical curricula [76,79,82,83]. It is necessary in this age to educate job or residency candidates about their digital voice and persona management [65]. Job or residency candidates should consider their publicly available web-based information as an extension of their resumé and should be aware that many employees use SM to investigate applicants. Therefore, candidates should ensure that their public SM profiles include nothing unprofessional about themselves [65,67]. Finally, the establishment of clear and equitable guidelines for searching candidates on SM is essential to prevent potential bias.

#### Professional Networking and Destressing

Professional connections represent important channels through which HCPs exchange knowledge, share expertise, refer patients, seek a second opinion, collaborate on research, hire and employ, provide social support, and improve health care outcomes. In the last few years, work-related communication has changed considerably with the advent of electronic communication tools, especially with the aid of instant messaging on smartphones [30,84]. Virtual professional communities can enable members to quickly access evidence-based information and disseminate work, which can lead to increased immediate impact [85,86]. Most SM platforms are found to be easy to implement, effective, quick, and low cost [87]. In a recent systematic review, positive predictors for using SM among HCPs were identified to be younger age, lower rank, and fewer years of experience, and the most commonly used platforms for communicating with colleagues were Facebook, Twitter, LinkedIn, and WhatsApp [88].

Owing to the exceedingly large number of HCPs on SM, platforms that are designed only for medical personnel have been introduced. Digital communication and social interactions occur between people who may or may not be known to each other [44]. In addition to medical issues, discussions usually address diverse subjects such as politics, practice management, career enhancement opportunities, and even dating in a medical environment [2,89]. Sermo, the world’s largest virtual doctors’ lounge, is a leading social network for physicians that is now available in 30 countries [90]. Doximity is a newer physician-only social networking platform with more than 500,000 members as of 2020 [91]. In addition, there is the Medical Directors Forum, which is an SNS exclusively for medical directors that provides a secure environment for peer-to-peer interaction [92]. Studies on HCPs’ preferred SM platform showed that Facebook was used most frequently (86%). Other commonly used platforms were Medscape Physician Connect (52%), Sermo (44%), LinkedIn (42%), YouTube (40%), Blogging (25%), and Twitter (20%) [26]. These statistics have been confirmed in subsequent studies [32]. For health-related reasons, physicians primarily used LinkedIn (70.7%) and Twitter (51.2%) [32]. Another study reported that HCPs spent an average of 11 to 13 hours per week on medical professional networking sites [26].

WhatsApp has been used as an intradepartmental, patient-related communication method because of its instant and more efficient handovers [93]. When physical proximity was a barrier, physicians preferred to use WhatsApp to exchange work-related knowledge over traditional text messages [84]. The American Academy of Pediatric Dentistry has a private group on Facebook with fewer than 3800 members who share clinical cases, clinical experiences, research results, new products, and relevant events [9]. Facebook has many other groups for dentists that are open to the public to view and join [30]. Microblogs such as Twitter allow a dynamic and concise exchange of information that is instantly accessible by an increasingly large number of readers [89]. Furthermore, the dissemination of scientific literature on SM (eg, Twitter) has increased the number of citations and downloads of published articles [94-96]. LinkedIn serves as a professional space for HCPs to demonstrate their expertise and capabilities; 54% of physicians have used it to communicate with colleagues [32,42].

SM also has a positive impact on students. The sense of belonging is crucial for undergraduate training. By being part of a well-respected learning environment, students benefit educationally and socially [97]. Moreover, having guidance and support on a 24/7 basis can ease their transition from university to more independent training centers [30].

In recent months, SM platforms have become helpful in maintaining communication with friends and family and reducing isolation and sense of loneliness, which could have a negative psychological impact [98]. Amid the COVID-19 pandemic, many practices have been affected and many jobs have been lost worldwide [99]. The sense of unity and comradery introduced by SM among users has helped countless individuals overcome hardships, including HCPs. In the first half of 2020, HCPs were deployed into unfamiliar environments because of the COVID-19 pandemic, worked beyond their areas of expertise and over long hours, and had to involuntarily isolate themselves from their families. This crisis has been anxiety inducing and stressful for HCPs, who often resorted to SM to voice their frustrations, experiences, and opinions not only with family and friends but also with the global network of frontline staff enduring similar challenges. The unique virtual siblinghood united the global health care community like never before. A Facebook page was created to facilitate the renting of recreational vehicles for HCPs to self-isolate outside their homes [100]. The public played an important role in paying due respect to HCPs, who were often referred to as heroes, both literally and figuratively. Illustrations portraying their pivotal role were shared on every known SM platform. Videos showing countless people applauding for HCPs at certain hours of the day went viral. Many HCPs engaged in what was labeled as *COVID-19–free zones* to escape, even if momentarily, from the pandemic stress. Clinicians from all specialties in diverse locations joined forces against a single enemy. Their voices echoed louder when they addressed lawmakers demanding improved access to personal protective equipment (PPE), increased testing for COVID-19, reduced reimbursement barriers to telemedicine, and improved mental health care.

To conclude, SM plays an important role in the lives of HCPs at a personal level. Whether SM is used for amusement, *zoning out*, or commiserating, it provides a safe haven for HCPs to put off their metaphoric heroes’ capes and find comfort in their humanhood again. Future research should investigate the role of SM in helping HCPs individually and collectively tackle the challenges resulting from the COVID-19 pandemic.

#### Professional Medical Education

Millennial students of health professions are increasing in numbers each year. They possess qualities consistent with being lifelong learners [71]. As Prensky [10] discussed, traditional education systems are no longer suitable for contemporary students. Millennials and younger generations process information in a fundamentally different manner from their predecessors. SM offers formal and informal educational opportunities and has the ability to remove physical barriers that could otherwise impede access to educational resources [51]. Not only is SM rich in educational resources but coupling the information with the interactive exchange of ideas and the live discussions has also made it a valuable educational tool. When SM was integrated into teaching, students were motivated by content obtained from SM, and positive behavioral changes were promoted [101]. Moreover, when SM was incorporated into clinical education, students perceived better collaboration with their peers, improved professional and career development, and larger supportive learning communities [102].

Social networks are an underutilized educational resource, not only for trainees but also for experienced clinicians. A large array of educational material is abundant on SM from seniors with advanced experience and from fellow trainees as well, usually at no cost to the user [30]. The differences between experts and novices are slowly diminishing because of novel forms of peer learning and knowledge production facilitated by SM [35]. Most platforms are frequently used to engage learners. YouTube in particular is more commonly used to teach technical skills and has been acknowledged by dentists as a convenient educational platform [30,71,103,104]. YouTube can also be used in classrooms to forge discussion, illustrate a procedure, or reinforce information, which promotes critical thinking and problem-solving skills [70]. A study conducted in Saudi Arabia found that YouTube was the most commonly used SM platform in medical education [105].

Evidence suggests that SM has a place in health care education. Universities use SM to create virtual classrooms and increase access to academic libraries [70]. In the United States, 95% of medical schools have some Facebook presence, and 71% of them have student groups [106]. In a study on nursing students, Twitter was used to view videos of clinical scenarios, and students tweeted their observations for instructor feedback [70]. Internet and SM content have been successfully used to train older caregivers to improve the caregivers’ and patients’ quality of life [5]. In a unique experience, the University of Rhode Island managed to connect students to geriatric patients on Facebook. It promoted students’ empathy and communication skills while helping patients advance their SM skills to battle loneliness [2]. In addition, students in an oral and maxillofacial radiology course perceived using Twitter as a helpful learning tool that enhanced access to faculty [107]. Moreover, medical students and professionals in cardiology reported the use of social networks for education and professional training [103].

Learning may be considered a social activity [106]. The more senses the students stimulate in their learning process, the more likely the information acquired is to be retained. Thus, the greater the engagement and contribution of the students, the better the learning outcomes. SM provides a medium for active collaboration rather than passive learning. In nursing, 92.4% of students perceived a positive learning impact from the podcasting of lecture materials [108]. In another study, students who were more heavily engaged in blog-based discussion of relevant learning material had higher grades than peers who had contributed less to the discussion [109]. Passing an examination was significantly associated with combining discussion on a WhatsApp group with the web-based question bank, and so were their higher grades [110]. Medical students who used Wikipedia had superior short-term knowledge acquisition compared with those who used a digital textbook, which suggested a potential role for Wikipedia in medical education [111]. In addition, medical students who integrated the use of SM in 2 elective courses were satisfied with the new approach [112]. Twitter and Instagram have also been described as helpful tools in radiology education [113]. Participants in a study reported that SM was perceived as helpful and very helpful for improving knowledge, creativity, decision making, critical skills, and problem-solving abilities [114].

In a study by Alsuraihi et al [105], YouTube, Facebook, and Twitter were among the most commonly used resources for learning. Although 95.8% of the students believed that SM was beneficial for learning, 40% thought it might be distracting [105]. In a review by Chan et al [85], it was found that multiple residencies used SM to broaden the horizons of trainees and facilitate engagement in journal clubs using virtual classrooms; a wide range of SM platforms were featured, including Facebook, blogs, Wikipedia, and podcasts. Specifically, dermatologists in a study agreed that WhatsApp discussions enriched their scientific knowledge of clinical cases and promoted learning about relevant references and upcoming meetings [115]. Participants of a 2015 study on surgical teams expressed that WhatsApp helped to flatten the hierarchy among students, residents, and experienced consultants, enabling them all to actively contribute to discussions without inhibition. This comfortable environment is especially helpful for shy and marginalized students [116].

Once students move beyond structured, supervised learning environments, they must recognize their own gaps in knowledge and skills over time and make every effort to fill them, adopting skills for lifelong learning [106]. The increasing mutability of knowledge in the digital age and its exchangeability and accessibility on mobile phones make learning thorough SM platforms a common practice for many medical students [35,117]. However, learning cannot be done through SM alone but is used to augment learning from textbooks, peer-reviewed research publications, and mentors, and just like with other sources of information, critical appraisal to information retrieved from SM must be applied; this is what lays the foundation for a future competent web-based learner [85,106,118]. It is important that students understand that educational material shared through SM cannot be accepted as is without a great degree of skepticism and objective evaluation.

To summarize, today’s students are unique in how they learn and acquire skills. Current educational systems must adapt to the needs and qualities of modern students and augment, if not replace, the traditional teaching methods with more digital means. It is essential for educators to put every effort in determining the best means of presenting information to their students and guiding them in their information search and appraisal. Retrospective research can be planned to compare the performances, learning outcomes, and teaching strategies between 2 student cohorts: those that were taught in a traditional manner and those that relied on SM.

#### Telemedicine

As people are becoming increasingly fluent in using novel technologies, health care has recently changed when, where, and how patients and HCPs communicate [119]. Telemedicine is the use of communication technologies and electronic information to provide health care support to patients or health care workers who are physically distant from HCPs [120]. Many branches of medicine are now adopting electronically mediated care; terms such as teledentistry and telepsychiatry are not uncommon, and publications related to telemedicine have been increasing [121-123]. Among the specialties that use telemedicine are pediatrics, psychiatry, diabetes, dentistry, nursing, palliative care, and allergies [124-130]. HCPs can now overcome their limited clinical time by communicating with their patients remotely. With the aid of novice technology, they provide a more convenient type of care for patients, especially for following up patients with chronic health care needs [131].

As young and highly qualified HCPs prefer staying in urban communities, telemedicine significantly augments clinical care, especially in underprivileged and underserved communities in rural areas [2,82,132,133]. Furthermore, as health care costs continue to increase, organizations are aiming to reduce costs without jeopardizing the quality of care being provided [134]. Recruitment and workforce sustainability are often an issue, and some countries with large, sparsely populated rural areas have grappled with how to overcome medical and dental provider shortages in these rural areas. Telemedicine and teledentistry can be of great use to ensure that new practitioners appointed in rural locations are not secluded and have the advice and support they need to promote their clinical work and psychological well-being [135]. It may also be used to connect HCPs in third-world countries with specialists in more medically advanced regions; for example, surgical procedures may be streamed live, and questions can be asked in real time [69].

Smartphones are fast, portable, and simple to use; mobile apps now seem to be ideal for quick learning or communication between colleagues or HCPs and the public or patients. Mobile apps are among the most commonly used tools for telemedicine [82]. Globally, but particularly in low- and middle-income countries, communication among HCPs is facilitated via WhatsApp, providing faster diagnosis and immediate management of acute findings [136-138]. A systematic review on telehealth concluded that 74% of the studies reported economic benefits of eHealth interventions for different medical conditions [134].

Programs for electronically monitoring intensive care units allowed HCPs to remotely monitor the conditions of up to 100 patients in multiple hospitals [139]. Pandemics and natural disasters pose challenges to effective and prompt health care delivery. Although telemedicine and eHealth might not solve them all, they can aid HCPs in providing the necessary management in scenarios in which the infrastructure is intact. In recent weeks, the distant triage that allowed patients to be efficiently screened for COVID-19 was patient centered and in compliance with self-quarantine; thus, it protected patients, clinicians, and the community from exposure to the virus [139]. For instance, replacing scheduled office visits with telemedicine visits in case HCPs were quarantined, absent, or sick was a productive initiative at Jefferson Health, Philadelphia, Pennsylvania [139]. An interesting model of telemedicine was explained by Baker and Stanley [40], in which patients use an app to navigate to a specific medical site, answer a few triage questions about their medical condition, wait in a virtual queue to be connected by video to an HCP, and discuss their condition or concern.

Sending clinical photographs privately between colleagues for a second opinion or to enrich discussion is not uncommon. A comprehensive review by Boulos et al [35] shares findings from multiple studies on the use of WhatsApp and Instagram in those contexts. One study found excellent inter- and intraobserver agreement in the assessment images of tibial fractures using WhatsApp [140]. There was a report of a life-saving use of WhatsApp in a resource-limited situation in which the life of a critically ill patient was saved by sending clinical monitor images with electrocardiogram changes and receiving feedback from an expert consultant who was 40 km away from the center where the patient was admitted [141]. Moreover, evaluating maxillofacial computed tomography scans using WhatsApp has been reported to be easy and rapid [142]. WhatsApp was also useful for communication between emergency department consultants when they were not onsite [143]. In dentistry, a study showed that 67.32% of dentists used WhatsApp to send clinical images to colleagues seeking second opinions, and 35.29% of them did so on a weekly basis. About 60.29% of the dentists received a prompt response, whereas 38.23% received delayed responses. In addition, about 98.52% of dentists sent radiographs on WhatsApp for a second opinion [144].

In conclusion, it is noteworthy that telemedicine is not *a practice in and of itself* [145]. It is not the most suitable model of care for every patient and is not the preferred approach when physicians cannot meet clinical standards of care. Patients using telemedicine must also have access to traditional emergency care, if needed. Although these innovations have significant potential benefits, the electronic exchange of health information and care may pose risks to patients’ privacy, confidentiality, and safety and to quality and continuity of care. Furthermore, the limitations of electronically mediated physical examination may weaken the relationship between patients and HCPs, thereby jeopardizing care [119]. High-quality research is needed to improve the utilization of telemedicine, and more well-designed studies comparing telemedicine with traditional patient care are essential.

#### Scientific Research

The perceived benefits of using SM in health care include the ability to connect with geographically distant researchers and to build and foster research communities [4]. SM is a potential tool to revolutionize health research, as it has fewer temporal and spatial limitations and can overcome boundaries between research communities and the public [146,147]. SM can aid research in several ways: by recruiting participants, disseminating surveys, connecting with fellow researchers, identifying research opportunities, sharing study findings, and gaining access to published work.

There are conveniences in taking scientific research to the digital world. Publishing study findings on SM provides enhanced dissemination of research and increases the access to valid evidence-based information for patients. Furthermore, because not all studies end in a publication in a traditional journal, their findings can thus be shared via SM to a wider audience and be of substantial value to a broader research community [4]. Another advantage of SM for scientific research became evident during the COVID-19 pandemic, which made it possible to break geographical barriers and arrange collaborative research projects, surveys, and multicenter studies [8]. Sites such as Google Scholar and ResearchGate create communities for researchers to network, collaborate with each other, and promote publications [53,148]. The anonymity of posts, not having to answer questions in the presence of others and acquiring large samples that attenuate the effect of false information or extreme views were viewed as advantages unique to SM surveys and possible factors that improve research accuracy [146]. Content posted on the web may be used as data for research without interacting with the authors of the content, and perhaps without even considering them to be *human subjects* [146]. Moreover, compared with traditional recruitment methods, web-based surveys have the ability to store large numbers of responses, which can be easily accessed for analysis [5].

Recruiting research participants on SM has gained popularity in recent years. In a review by Lafferty and Manca [4], it was found that the most common tools used for recruiting participants were Facebook, Twitter, and a combination of both. Snowballing sampling method involves participants themselves recruiting more participants by contacting people in their networks [149]. A study on 8252 participants found that web-based recruitment was more efficient and had lower costs per recruited participant compared with traditional methods [150].

Disseminating surveys on the web is now a common practice. One study chose SM platforms to send its survey because it was cost effective, time saving, and easily accessible [151]. In another dentist or patient study, the survey for dentists was distributed via a dentist-only Facebook group that had more than 4500 members; for patients, the survey distribution was mainly through Facebook, LinkedIn, and Twitter, and the recipients were asked to share it with their connections [42]. Furthermore, in a study involving health care quality personnel, the survey was distributed through WhatsApp [114]. In a study in Saudi Arabia, the link to the web-based questionnaire was made available through Twitter and Facebook, the 2 most popular SNSs in the country [152]. Over half of university students strongly or somewhat liked using Facebook for research conducted by university researchers [153]. Zaballos et al [154] developed a web-based multiplatform that integrated WhatsApp and emails to assess the quality of life of individuals with hearing loss issues; the tool facilitated data collection in an easy-to-use platform.

A review by Topolovec-Vranic and Natarajan [155] showed that 40% of the studies found SM to be the most effective recruitment method, whereas 50% of them stated that their target population was *hard to reach*. Approximately 43% of the studies reported cost-effectiveness [155]. In addition, SM helped in recruiting a large number of individuals and reached challenging populations such as adolescents and young adults. Another review found that traditional recruitment methods tend to underrepresent users of marijuana, ecstasy, cocaine, or alcohol or people with at-risk sexual behavior; in comparison, Facebook recruitment yielded more representative results [156].

Researchers who plan to recruit participants on SM must consider their target populations’ SM use patterns and preferences. For example, a study on sexual health might consider dating sites for recruitment, whereas Facebook may be more suitable for a nonsexual health study [157]. To best tailor recruitment campaigns, the selection of hashtags or keywords that reflect the interests of the target population might be useful [45].

Regarding shortcomings, it is important to note that research participants recruited from web-based environments may not truly represent the population of interest as a whole, suggesting that SM should only augment traditional recruitment methods [4]. A study suggested that people with disabilities may disproportionately be living in conditions with lower standards of living and may not have access to the internet [158]. In another study, subjects recruited from SM were largely middle class, whereas those recruited at a local hospital were more disadvantaged [159]. A review by Whitaker et al [156] showed an overrepresentation of young White women resulting from web-based recruitment.

Other limitations of using SM for research include that researchers have little control over distractions, the research idea may be copied, or participants may share research information with other participants, which puts the scientific integrity of the study at risk [4]. In a review by Denecke et al [5], the most reported ethical concerns for using SM for research recruitment were self-selection—that is, users with an interest in the study area will be recruited preferentially, which will affect the representativeness of the sample—and a skew toward well-educated and higher socioeconomic status cohorts on the web [5].

Ethical and privacy concerns regarding SM for research recruitment must be addressed because tracking, profiling, and targeting of users are common in the digital world [45]. Bender et al [160] proposed privacy-enhanced SM recruitment guidelines, including proactive measures to protect privacy and declaration of potential risks. Vulnerable groups such as children and teenagers, homosexuals in regions where homosexuality is illegal, and individuals with mental illnesses require extra emphasis on respect, confidentiality, and caution in obtaining consent [146].

To summarize, there is growing evidence to suggest that SM is a useful research tool that enables researchers to connect with each other, recruit participants, and share their findings with the public. Moreover, the data obtained from SM can be investigated. Nevertheless, researchers must not overlook the shortcomings of SM that may ultimately debilitate the integrity of the study. Privacy concerns and ethical considerations must also be considered. The development of guidelines for ethical conduct in web-based research should be based on the best available practices and should be comprehensive and standardized to minimize a study’s error margin. Future studies that compare different recruitment methods and varying participant demographics recruited using various methods should be encouraged. Research investigating the cost-effectiveness of SM research and those with large sample sizes that enable the generalizability of findings is also recommended.

#### Critical Public Health Care Issues

SM can be used by emergency notification systems to mass communicate information to large groups in a fast and low-cost manner. Studies have shown that SM can be a source of data to detect outbreaks, infection distribution, and areas of acute health care needs [29]. It can also help understand the public’s knowledge, fears, attitudes, and behaviors during a crisis [161-163]. For example, the Red Cross tracks Twitter posts during natural disasters, such as hurricanes and earthquakes, to assess where the greatest needs lie [50,164]. Perhaps one of the first publications investigating SM use during a pandemic is a study that analyzed tweets posted during the 2009 H1N1 outbreak; this study found that SM can be a useful tool for disseminating information and for the public to share their opinions and experiences [165]. Twitter posts were also helpful in monitoring disease activity during the cholera and influenza outbreaks [166,167]. When interaction and collaboration were essential, as with the influenza A-H1N1 pandemic, SM provided an unmatched opportunity to engage the public and was used by prominent health organizations such as the WHO [7,41]. However, coordination between web-based and real-world response activities is also important [29].

Perhaps there is no more powerful example of SM use during a health crisis than what has happened during the COVID-19 pandemic. The dissemination of information during a pandemic has never been this quick and effective in the past. Information on the virus spread as quickly as the virus itself and dominated conversations on SM. On March 11, 2020, there were more than 20 million mentions of coronavirus-related terms on SM [168]. Since the beginning of the outbreak, SM has been one of the most commonly used communication channels by international health organizations such as the WHO and the CDC to possibly disseminate information to every person on earth with access to SM. Thousands of smaller health authorities may have also used SM to communicate with local communities. Although traditional access to medical guidelines and policies often requires some form of affiliation or membership, it is available to internet users today with a tap on a keyboard or a finger slide on a smartphone. The distribution of PPE, sharing treatment protocols, clinical trial results, and allocation of medical resources have been efficient with the aid of SM [8]. A recent study evaluated the 100 most viewed *coronavirus* videos on YouTube; as of March 5, 2020 (very early in the pandemic), these videos had 165 million views [169]. Another study in China collected data from 250 million Weibo users, a Twitter-like SM platform. Posts mentioning symptoms or diagnoses significantly predicted daily case counts ahead of the statistics announced by officials in Hubei Province, the epicenter of the initial outbreak, and the rest of China [170].

Perhaps the founders of Twitter did not expect it to become a helpful tool in the fight against COVID-19. For example, using Twitter, a cardiologist was able to expedite the delivery of a drug to a COVID-19 patient within just 6 hours of his tweet [171]. The American Heart Association launched a registry on Twitter to aggregate COVID-19 cases to better understand risk factor profiles and treatment algorithms [171]. Hashtags such as #GetUsPPE highlighted the scarcity of PPE, resulting in technology pioneers ramping up their production of PPE [171]. After calls were raised on Twitter and other SM platforms, HCPs flew to other parts of their countries that were in crisis, retired clinicians volunteered to rejoin the work force in several countries, and those who were unable to be present helped colleagues through telemedicine. Another example of SM use during the pandemic is the COVIDBRONCH Initiative—an international network of airway specialists who foster rapid acquisition and dissemination of knowledge regarding airway procedures during the pandemic [172].

Despite its catastrophic impact and the substantial loss of lives, humans will overcome the existential threat brought by COVID-19 and will also likely overcome future pandemics. Over time, humans have survived environmental, biological, and man-made calamities because of their innate adaptability, resilience, innovativeness, and persistence. Today, humans use SM to disseminate information quickly and to a large number of people, thus eliciting an almost immediate response. More research is already taking place and will continue to investigate the key role of SM in the fight against pandemics, not only from a medical perspective but also from social and economic viewpoints.

### Conclusions

This review provided an overview of the different uses of SM in health care. It is evident that SM use indicates not a trend but a fundamental shift in how people communicate today. Multidimensional health care, which includes SM and other forms of communication, has been shown to be highly successful. Not only can SM be used to improve direct patient care but it can also be used to increase the public’s knowledge, facilitate research, connect HCPs, improve medical education, and combat public health crises. However, striking the right balance between digital and traditional health care is imperative. As SM is a relatively recent phenomenon, further research is needed to determine its long-term effectiveness and to identify the best strategies for maximizing its advantages and limiting risks. This review will be continued in the second part, in which the use of SM from patients’ perspectives will be discussed. This discussion will be supplemented with specific barriers, ethical considerations, and disadvantages reported in the extant literature.

## Abbreviations

CDC: Centers for Disease Control and Prevention
HCP: health care provider
PD: program director
PPE: personal protective equipment
ROI: return on investment
SM: social media
SNS: social networking site
WHO: World Health Organization
